# Electrochemical Performances Investigation of New Carbon-Coated Nickel Sulfides as Electrode Material for Supercapacitors

**DOI:** 10.3390/ma12213509

**Published:** 2019-10-25

**Authors:** Xinyu Lei, Mu Li, Min Lu, Xiaohui Guan

**Affiliations:** School of Chemical Engineering, Northeast Electric Power University, Jilin 132000, China; leixinyu_k@163.com (X.L.); limu910308@163.com (M.L.); lumin19770919@163.com (M.L.)

**Keywords:** nickel sulfides, carbon nanotubes, carbon-coated, supercapacitors, electrode material

## Abstract

A new carbon-coated nickel sulfides electrode material (NST/CNTs@C) has been synthesized through an easy-to-operate process: NiS_2_/CNTs which was prepared by a hydrothermal method reacted with BTC (1,3,5-benzenetricarboxylic acid) under the condition of water bath heating to obtain the precursor, and then the precursor was calcined in 450 °C under a nitrogen atmosphere to obtain NST/CNTs@C. The electrochemical performance of NST/CNTs@C has been greatly improved because the formation of a carbon-coated layer effectively increased the specific surface area, reduced the charge transport resistance and inhibited the morphological change of nickel sulfides in the charge–discharge process. Compared with pure NiS_2_ and NiS_2_/CNTs, NST/CNTs@C presented great specific capacitance (620 F·g^−1^ at a current density of 1 A·g^−1^), better cycle stability (49.19% capacitance retention after 1000 cycles) and more superior rate capability (when the current density was raised to 10 A·g^−1^ the specific capacitance remained 275 F·g^−1^).

## 1. Introduction

Nowadays, with the problems of global warming, fossil energy shortage and environmental pollution, developing and improving the utilization rate of renewable energy has become a research hotspot [1]. Therefore, it is necessary to enhance the storage capacity of existing energy storage devices. Supercapacitors (SCs), as some of the most promising energy storage devices, have received extensive attention, due to their high power and energy density [2], long cycle life [3] and being environmentally friendly [4]. SCs has already been widely utilized in many fields including consumer electronics [5], vehicles [6] and wearable devices [7]. As is well-known, the electrode materials are the most significant parts of supercapacitors to achieve charge storage [8]. On the basis of the energy storage mechanism of electrode materials, SCs can be classified as two types: (1) electrical double-layer capacitors (EDLCs) with carbon materials as the electrode materials; (2) pseudocapacitors (PCs) with transition metal compounds and conducting polymers as the electrode materials [9]. Normally, transition metal compounds have higher specific capacitance and energy density than carbon materials and conducting polymers because they not only store energy like carbon materials using double-layer but also occur fast and reversible Faradaic redox reaction [10,11].

In recent years, various transition metal compounds, such as NiO [12], MnO_2_ [13], Ni(OH)_2_ [14], Co(OH)_2_ [15], CuS [16], NiS [17], and NiS_2_ [18], have been studied as electrode materials for supercapacitors. Particularly, nickel sulfides (NiS, NiS_2_ and Ni_3_S_2_ et al.) which are easy to synthesize [19] with multiple valences [20] and low cost [21] have great application potential in supercapacitors. However, nickel sulfides also have some disadvantages, such as low rate capability and poor cyclability [22,23,24]. Lu etc. prepared NiS_2_ hollow spheres with specific capacitance up to 1643 F·g^−1^, but after 1000 cycles, the specific capacitance retention rate was only 27.7% [25]. Yang and his co-workers reported flower-like β-NiS had a specific capacitance of 857.76 F·g^−1^ at a current density of 2 A·g^−1^, but the specific capacitance sharp decreased when the current density raised to 5 A·g^−1^ [26]. The poor cyclic stability and low rate capability limit the practical application of nickel sulfides.

To solve these problems, various nickel sulfide composites have been constructed by combining them with different carbon materials such as carbon nanotubes (CNTs) [27], graphene [28], carbon-coated layer [29], and carbon fiber paper [30]. These carbon materials are usually mixed with transition metal compounds through different patterns, depending on the synthetic methods and types of materials. CNTs as one kind of electrode material has good chemical stability, low mass density, high conductivity, and large surface area [31,32]. Some previous reports have confirmed that the combination of transition metal compounds and CNTs can indeed improve the specific capacitance [33,34]. Moreover, Carbon-coated method can effectively improve the conductivity and mechanical strength of electrode materials, which is conducive to amelioration of electrochemical stability and rate capability. For example, Li’s group constructed V_2_O_3_ nanoflakes@C composites using activated carbon as carbon source, the rate performance of it was higher than pure V_2_O_3_ nanoflakes [35]. Shao et al. synthesized NiCoOP@C by hydrothermal process, carbonization and phosphorization treatment. Compared with NiCoOP without carbon-coated, the specific capacitance retention increased from 57% (NiCoOP) to 100% (NiCoOP@C) after 3000 cycles [36]. Guo and his co-workers prepared MnO_2_@C composite nanorods choosing glucose as carbon source, not only the cyclic stability of the MnO_2_@C composite nanorods has been improved but also the charge-transfer resistance was much lower than that of pure MnO_2_ [37]. Common carbon sources include glucose [38], soluble starch [39], and sulfonated polystyrene [40]. In this paper, 1,3,5-benzenetricarboxylic acid (BTC) (Aladdin Industrial Corporation, Los Angeles, CA, USA.), a novel carbon source was adopted. BTC is often used as organic skeleton to prepare MOFs, because it contains plenty of oxygen-containing functional groups which has strong adsorption capacity for metal ions and can coordination with metal ions [41,42]. However, using it as carbon source for carbon-coated reaction has rarely been reported. In order to compare the effect of different carbon-coated materials on electrochemical performance of nickel sulfides. BTC, polyaniline (PANI) and glucose were selected as carbon sources. The carbon-coated reaction processes using PANI and glucose were based on the methods of the previous references [43,44]. It can be seen that the specific capacitance, cyclic stability and rate performance of electrode material which used BTC as carbon source were the highest in Table 1. It indicated that using BTC as carbon source had certain advantages.

**Scheme 1:**
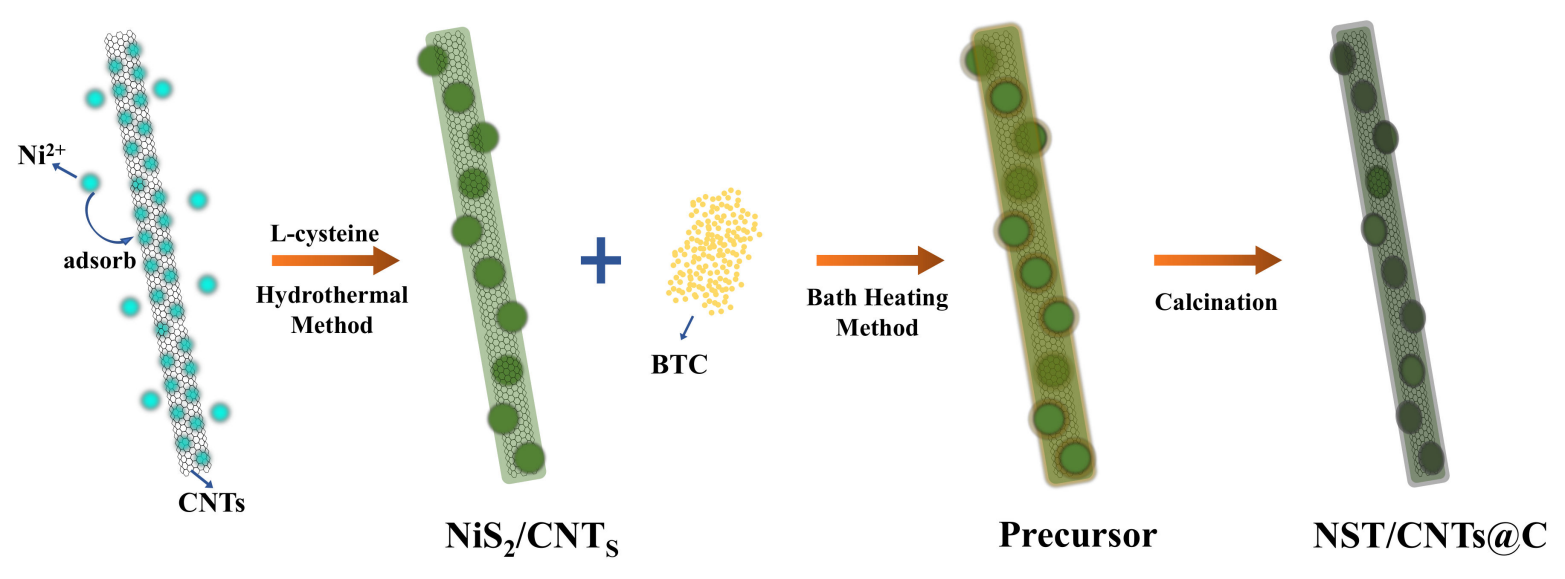
Schematic illustration of the synthetic route of NST/CNTs@C.

Herein, the purpose of our design was to simultaneously improve the specific capacitance and cycle stability of nickel sulfides. CNTs and carbon-coated materials played different roles; the former was introduced to increase the specific capacitance, and the latter was used to further enhance the stability. Firstly, NiS_2_/CNTs was prepared by hydrothermal method. The surface of surface-modified CNTs had a mass of polar groups to adsorb Ni^2+^, and NiS_2_ crystallized and grown on the surface of CNTs. Due to synergistic effect between NiS_2_ and CNTs, the specific capacitance of NiS_2_/CNTs was greatly enhanced. Next, NST/CNTs@C was prepared by water bath heating method and calcination process. BTC organic molecules adsorbed and deposited on the surface of NiS_2_/CNTs and possibly happened coordination reaction with NiS_2_ in water bath heating process to obtain the precursor [45], and then the precursor was calcined in inert gases to carbonize. The formation of carbon-coated layer has successfully improved the rate performance and cycle stability. The synthetic route of NST/CNTs@C was shown in Scheme 1.

## 2. Materials and Methods

### 2.1. Materials

All of the chemical reagents that used in this experiment were analytical grade and without further purification. All of the solutions were formulated with distilled water.

### 2.2. Surface Modification of CNTs

A sample of 1.0 g raw CNTs (Multi-walled CNTs were purchased from SUSN Limited Company in Shenzhen, China, specific surface area: 85–110 m^2^·g^−1^, diameter: 20–50 nm.) were added into 100 mL of mixture that was constituted by concentrated H_2_SO_4_ and concentrated HNO_3_ (volume ratio of 3:1) with magnetic stirring for 10 min. And then the mixture was heated in water bath at 80 °C for 3 h. After the mixture was cooled to the room temperature, vacuum filter was used to filter the product with water and ethanol until the effluent became neutral. Finally, the product was dried in a vacuum drying oven at 60 °C for 24 h to obtain surface-modified CNTs which were pulverized with the agate mortar and pestle.

### 2.3. Preparation of NiS_2_/CNTs

A sample of 0.2908 g Ni(NO_3_)_2_·6H_2_O and 0.0909 g urea were dissolved in 60 mL of distilled water with magnetic stirring for 10 min, and then 0.0123 g surface-modified CNTs were added with ultrasonic treatment for 30 min to disperse evenly, next 0.3635 g L-cysteine was added and ultrasonic treated for 1 h until it was completely dissolved. After that, the solution was transferred into a 100 mL Teflon-lined stainless steel autoclave and subsequently kept at 140 °C for 16 h. After cooling down to the room temperature, the black product was collected and washed with distilled water and ethanol for several times to remove impurity ions by centrifugation. Finally, the product was dried in a vacuum drying oven at 60 °C for 24 h to obtain NiS_2_/CNTs. The same method was used to prepare NiS_2_ for comparison, just not adding surface-modified CNTs. 

### 2.4. Preparation of NST/CNTs@C

A sample of 0.2456 g NiS_2_/CNT_S_ were added into 40 mL of N, N-Dimethylformamide (DMF) with ultrasonic treatment for 30 min. Meanwhile, 0.1261 g BTC was dissolved into 20 mL of alcohol with ultrasonic treatment for 10 min. After that, these two solutions were mixed together and then the mixture was stirred continuously and heated in water bath at 60 °C for 12 h. After reaction, the black product was collected and washed with DMF and ethanol for several times to remove impurity ions by centrifugation. The product was dried in a vacuum drying oven at 60 °C for 24 h to obtain the precursor. Finally, the precursor was calcined in 450 °C for 2 h under nitrogen atmosphere at a ramping rate of 1 °C·min^−1^ to obtain NST/CNTs@C.

### 2.5. Characterization

In order to identify the phase composition and the valence states of samples, powder X-ray diffraction (XRD, XRD-7000, Shimadzu, Chiba, Japan) with a Cu-Kα radiation source and X-ray photoelectron spectroscopy (XPS, ESCALAB-250, Thermo Fisher, Waltham, MA, USA) with an Al-Kα radiation source were used. Scanning electron microscope (SEM, XL-30 FEG, FEI, Hillsborough, OR, USA.) and transmission electron microscope (TEM, TECNAI F20, FEI, Hillsborough, OR, USA) were used to investigate the structure and morphology of the samples. The specific surface area and pore structure were determined by N_2_ adsorption/desorption experiment (ASAP 2020 plus HD88, Micromeritics Instrument Corp, Norcross, GA, USA). Chemical bonding information of the studied samples was gathered with Fourier transformed infrared spectroscopy (FTIR, Nicolet iS50, Thermo Fisher Scientific, Waltham, MA, USA).

### 2.6. Electrochemical Measurements

In this study, Parstat 4000 electrochemical workstation was used to test the electrochemical performance of the samples at room temperature. Three-electrode system including the Hg/HgO electrode as reference electrode, the platinum foil as counter electrode and the working electrode were used. The working electrode was produced by pressing a slurry of active material, acetylene black and polytetrafluoroethylene (mass ratio of 8:1:1) onto the nickel foam (1·1 cm^2^), and the load of active material was controlled about 8 mg. 2.0 M KOH solution was adopted as electrolyte solution.

## 3. Results and Discussion

### 3.1. Materials Characterization

**Figure 1:**
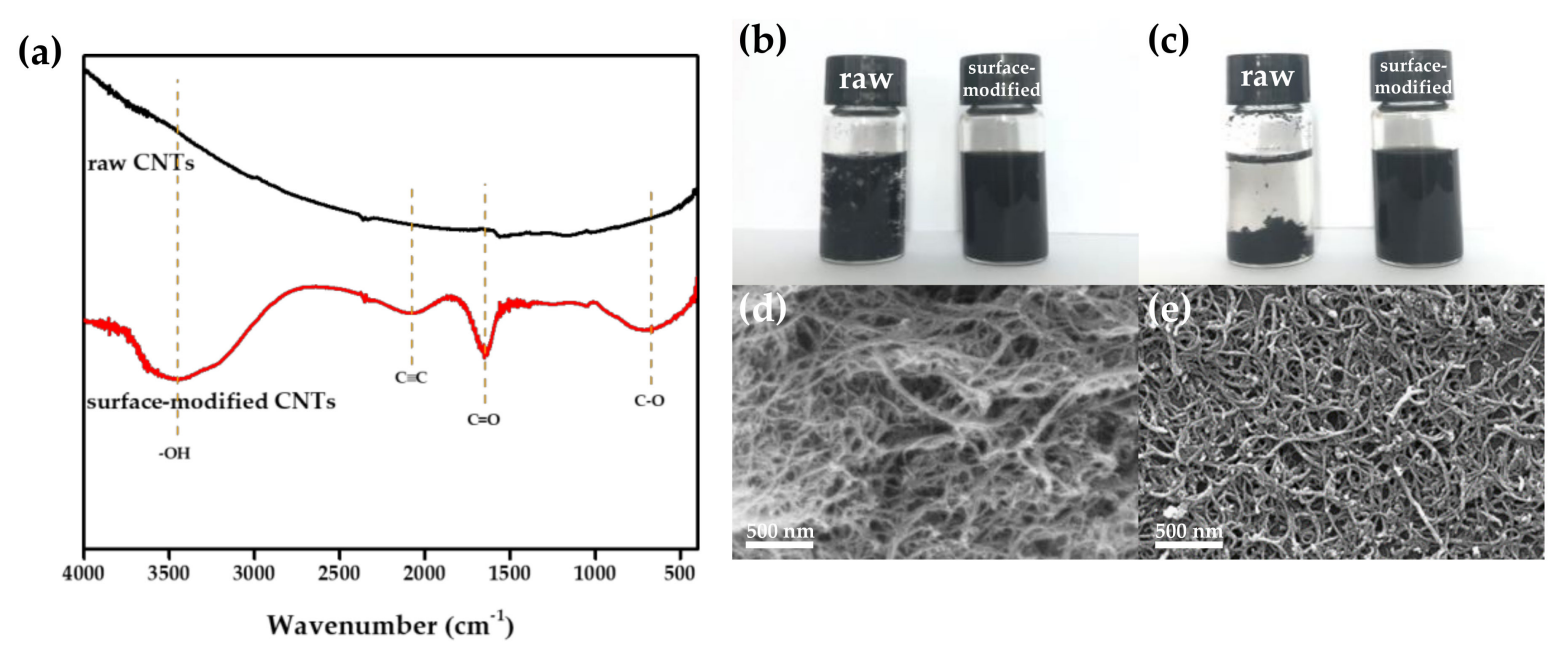
(**a**) FT-IR of the raw CNTs and the surface-modified CNTs; Dispersion state of the raw CNTs and the surface-modified CNTs in distilled water after standing for a certain time: (**b**) 0 h and (**c**) 24 h; (**d**) SEM image of the raw CNTs; (**e**) SEM image of the surface-modified CNTs.

Surface modification provided abundant oxygenated sites on the surface of CNTs and removed amorphous carbon and impurities [46]. Compared with the raw CNTs, the surface of surface-modified CNTs appeared vibrational peaks of –OH, C=O and C–O et al., (Figure 1a). These polar functional groups can improve the hydrophilicity and adsorption capacity of CNTs. The improvement of hydrophilicity not only enable CNTs to be better dispersed in distilled water but also increase the active sites on CNTs to adsorb Ni^2+^. As shown in Figure 1b,c, the surface-modified CNTs was more evenly dispersed in distilled water and still wasn’t sedimentation after standing 24 h. The diameter of CNTs that almost no changed before and after treatment was approximately 20–50 nm as shown in Figure 1d,e.

**Figure 2:**
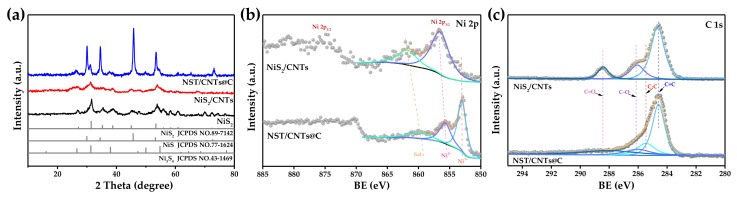
(**a**) X-ray diffraction patterns of three electrode materials; XPS spectra for (**b**) Ni 2p and (**c**) C 1s of NiS_2_/CNTs and NST/CNTs@C.

The phase compositions were studied by XRD, the results of NiS_2_, NiS_2_/CNTs and NST/CNTs@C were shown in Figure 2a. It showed that the peak positions of diffraction peaks of NiS_2_ and NiS_2_/CNTs were the same, both of which were in accordance with the standard data of cubic NiS_2_ (JCPDS No.89–7142). According to references [47,48], the reaction route for the synthesis of NiS_2_ could be expressed as the following chemical Equations:

(1) $${\mathrm{HSCH}}_{2}{\mathrm{CHNH}}_{2}\mathrm{COOH}+H_{2}O\to {\mathrm{CH}}_{3}\mathrm{COCOOH}+{\mathrm{NH}}_{3}+H_{2}S$$

(2) $$H_{2}{\mathrm{NCONH}}_{2}+3H_{2}O\to 2{\mathrm{NH}}_{4}^{+}+2{\mathrm{OH}}^{-}+{\mathrm{CO}}_{2}$$

(3) $$\mathrm{Ni}{\left({\mathrm{NO}}_{3}\right)}_{2}\to {\mathrm{Ni}}^{2+}+2{\mathrm{NO}}_{3}^{-}$$

(4) $${\mathrm{Ni}}^{2+}+2H_{2}S+2{\mathrm{OH}}^{-}\to {\mathrm{NiS}}_{2}+H_{2}O+H_{2}$$

The diffraction intensity of NiS_2_ was stronger than NiS_2_/CNTs because the addition of CNTs reduced the crystallinity. NiS_2_ happened phase transition during the calcination process, as the result of XRD analysis that NST/CNTs@C include two different phases: one was hexagonal NiS (JCPDS No.77–1624) and the other was cubic Ni_3_S_4_ (JCPDS No.43–1469). As the XPS spectra of Ni 2p shown in Figure 2b, NiS_2_/CNTs exhibited 856.49 eV binding energy which corresponded to the Ni^2+^. Meanwhile, the Ni^1+^ (853 eV) and Ni^2+^ (855.68 eV) were coexistence in NST/CNTs@C, indicating the characteristic of NiS and Ni_3_S_4_ [49,50]. Figure 2c revealed that carbon species had been changed, due to carbon-coated layer was formed on the surface of the raw material by carbon-coated treatment [51].

**Figure 3:**
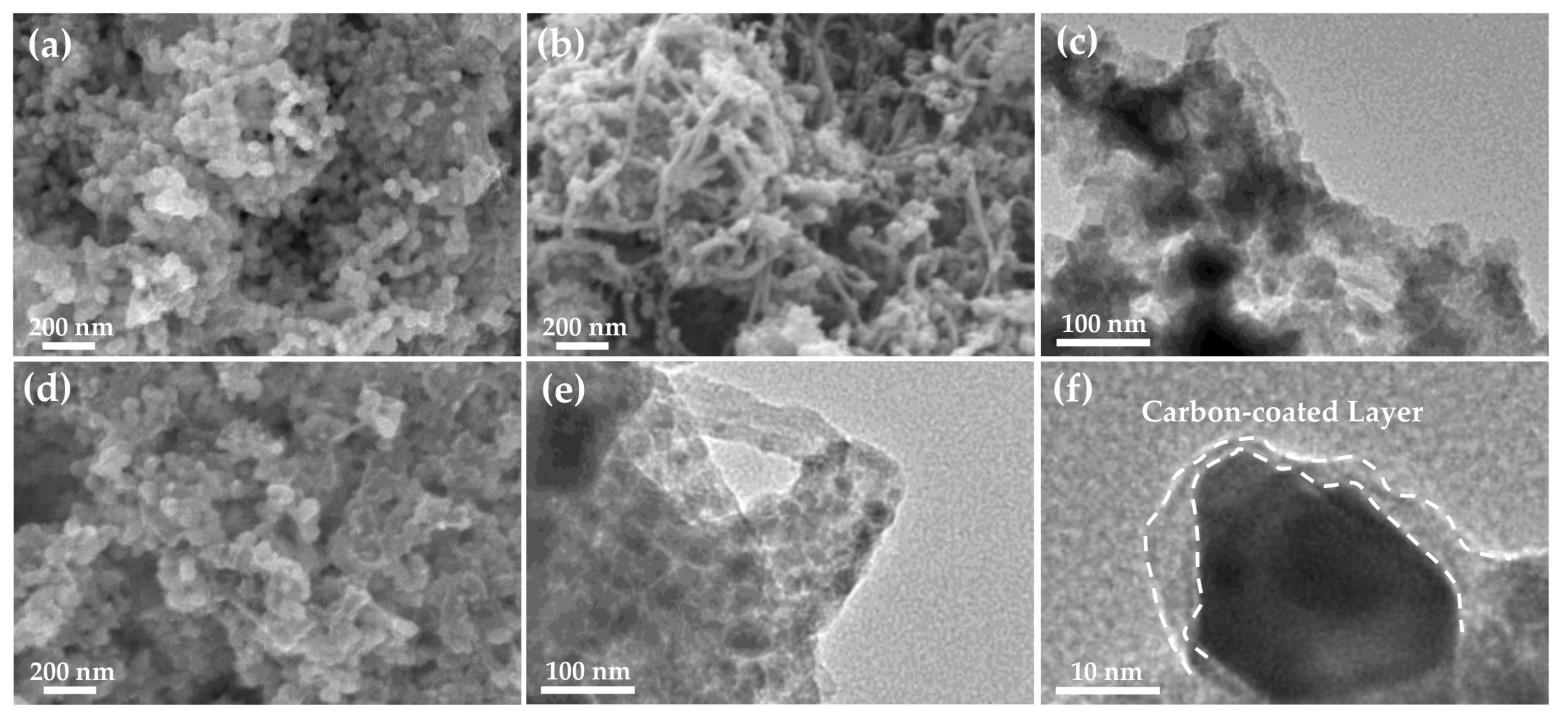
(**a**) SEM image of NiS_2_; (**b**) SEM image of NiS_2_/CNTs; (**c**) TEM image of NiS_2_/CNTs; (**d**) SEM image of NST/CNTs@C; (**e**) TEM image of NST/CNTs@C; (**f**) RHTEM image of NST/CNTs@C.

The morphology and microstructure of NiS_2_, NiS_2_/CNTs and NST/CNTs@C were investigated using SEM and TEM. It can be directly seen that the morphology of NiS_2_ was irregular nanoparticles whose surface was rough and diameter was approximately 20–50 nm, as shown in Figure 3a. From Figure 3b,c it can be seen that CNTs were coated with NiS_2_ and grown together with NiS_2_ nanoparticles. This kind of morphology of NiS_2_/CNTs nanocomposites were beneficial to enhance the specific surface area and reaction active sites, which may promote redox reaction and charge transfer [52]. The average diameter of nickel sulfides particles of NST/CNTs@C (Figure 3d) was a little larger than NiS_2_/CNTs, and it could obviously be observed that a carbon-coated layer was formed on the surface of NST/CNTs@C in Figure 3e,f. The carbon-coated layer could effectively limit the volume expansion of nickel sulfides in the charge–discharge process [53,54].

**Figure 4:**
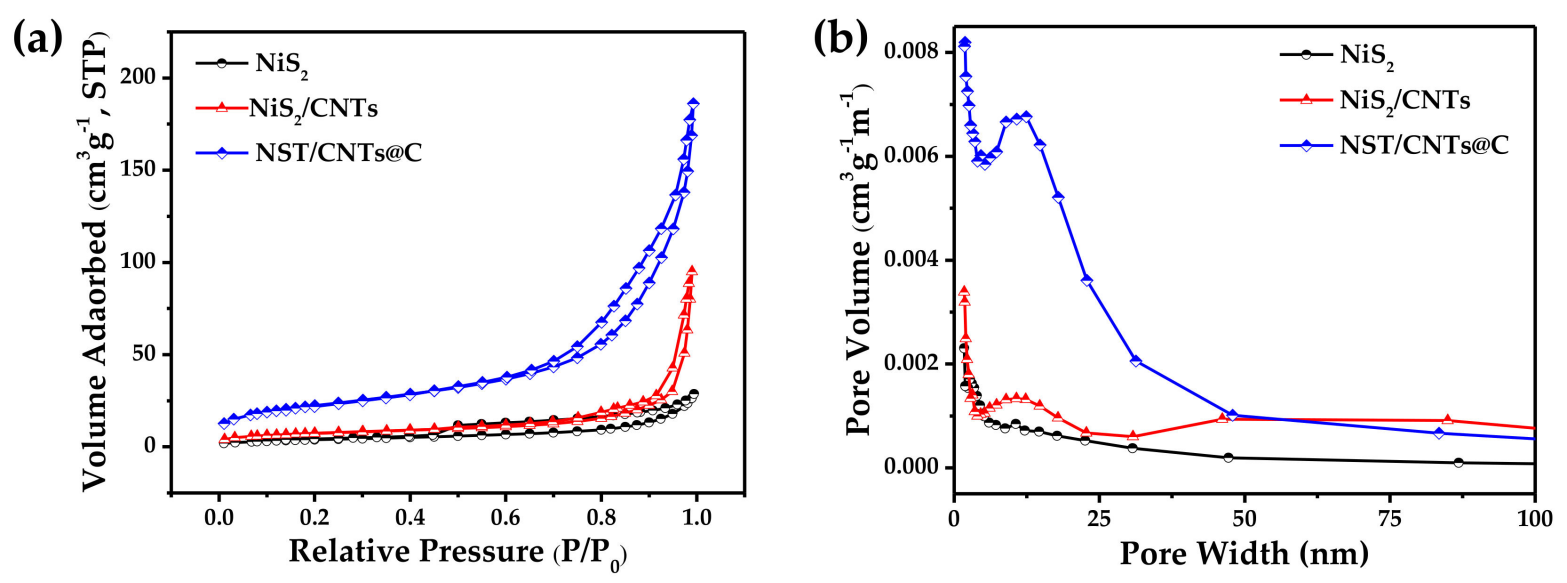
(**a**) N_2_ adsorption/desorption isotherms; (**b**) Corresponding pore-size distribution curves.

The specific surface areas and pore structures of NiS_2_, NiS_2_/CNTs and NST/CNTs@C were investigated using N_2_ adsorption/desorption measurement. The adsorption isothermal curves of three electrode materials were type IV adsorption isotherm which were showed in Figure 4a, and the curves revealed distinct hysteresis loops. It indicated that all three materials belong to mesoporous structure. The specific surface areas of NiS_2_, NiS_2_/CNTs and NST/CNTs@C were 14.39 m^2^·g^−1^, 25.80 m^2^·g^−1^ and 79.51 m^2^·g^−1^, respectively. By compounding with NiS_2_, the specific surface area of NiS_2_/CNTs had been improved. There were two main reasons: (1) CNTs has a larger specific surface area; (2) The introduction of CNTs improved the dispersity of NiS_2_. Moreover, the specific surface area of NST/CNTs@C had been greatly increased. This may be because the carbon-coated layer has larger pore volume (Figure 4b). Furthermore, A large specific surface area provided a higher number of reactive sites for electrode materials to improve the electrochemical properties.

### 3.2. Electrochemical Properties

**Figure 5:**
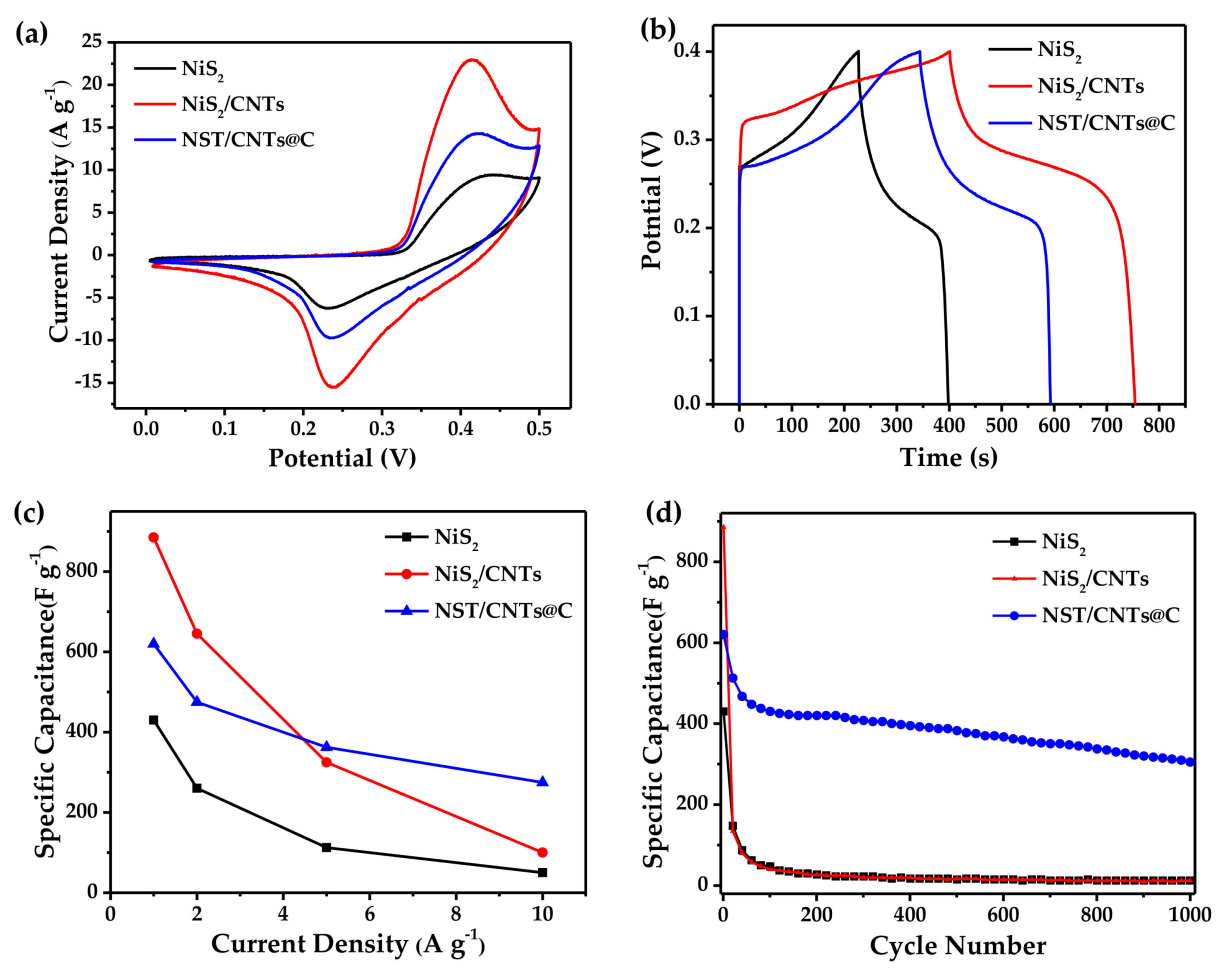
(**a**) CV curves at a scan rate of 10 mV·s^−1^; (**b**) GCD curves at a current density of 1 A·g^−1^; (**c**) Specific capacitance at various current density; (**d**) Capacity retention during 1000 cycles of GCDat a current density of 1A·g^−1^.

The cyclic voltammetry (CV) curves of NiS_2_, NiS_2_/CNTs and NST/CNTs@C at a scan rate of 10 mV·s^−1^ within the voltage window from 0 V to 0.5 V are shown in Figure 5a. The CV curves of three electrode materials all had paired redox peaks, which meant all of three kinds of electrode materials that possessed reversible redox reactions and revealed quasi-capacitance ability. Obviously, the CV curve of NiS_2_/CNTs presented the largest integrated area corresponded to the maximum specific capacitance. This conclusion was consistent with the consequence of galvanostatic charge–discharge p (GCD) tests shown in Figure 5b. The specific capacitance of NiS_2_, NiS_2_/CNTs and NST/CNTs@C were calculated according to the Equation (5) to be 430 F·g^−1^, 885 F·g^−1^ and 620 F·g^−1^ at a current density of 1 A·g^−1^, respectively.

(5) $$C_{m}=\left(I\cdot ∆t\right)/m∆V$$

where *C_m_* is the mass specific capacitance (F·g^−1^), *I* is the discharge current (A), *m* is the mass of the active material (g), *∆t* is the discharge time (s) and *∆V* is the voltage range (V), respectively [55]. The specific capacitance has been improved because of the addition of CNTs. Furthermore, the specific capacitance of NST/CNTs@C was lower than NiS_2_/CNTs but much higher than NiS_2_. It was due to the transformation from NiS_2_ into NiS and Ni_3_S_4_ during the calcination process, and compared with NiS_2_ (870 mAh·g^−1^), NiS (589 mAh·g^−1^) and Ni_3_S_4_ (703 mAh·g^−1^) exhibits an inferior theoretical capacity [56].

**Figure 6:**
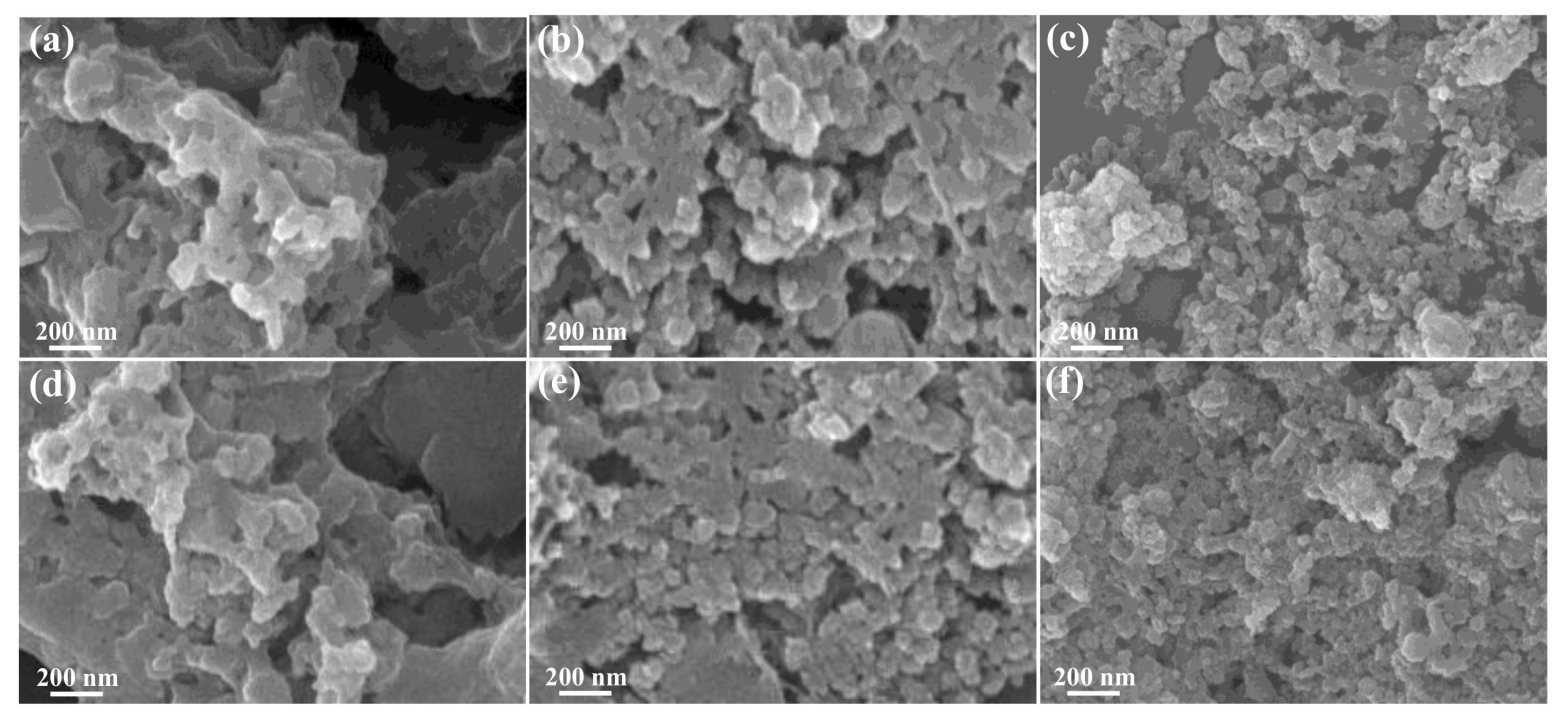
SEM images of (**a**) NiS_2_, (**b**) NiS_2_/CNTs and (**c**) NST/CNTs@C after 20 cycles; SEM images of (**d**) NiS_2_, (**e**) NiS_2_/CNTs and (**f**) NST/CNTs@C after 1000 cycles.

As shown in Figure 5c, when the current density was elevated to 5 A·g^−1^, the specific capacitance of NiS_2_, NiS_2_/CNTs and NST/CNTs@C declined to 112.5 F·g^−1^, 325 F·g^−1^ and 362.5 F·g^−1^, respectively. The specific capacitance of NST/CNTs@C was the highest of the three electrode materials. Moreover, while the current density continued to rise to 10 A·g^−1^, the specific capacitance retention rates of three kinds of electrode materials were 11.62%, 11.30% and 44.35%, respectively. The above results suggested that although carbon-coated treatment reduced the specific capacitance, it greatly improved the rate performance. Figure 5d shows the cyclic performance of these electrode materials at a current density of 1 A·g^−1^ and the voltage range was from 0 V to 0.4 V. The specific capacitance of NiS_2_ and NiS_2_/CNTs dropped sharply in the first 20 cycles, from 430 F·g^−1^ and 885 F·g^−1^ declined to 54 F·g^−1^ and 59 F·g^−1^, respectively. The rapid decrease of specific capacitance may be owing to the transformation of microstructure which was caused by the volume expansion and contraction in the process of redox reactions [57]. After 20 cycles, the morphology of NiS_2_ and NiS_2_/CNTs electrode materials were presented in Figure 6a,b. The changes of the morphology were enormous, dispersed NiS_2_ nanoparticles almost completely disappeared and only the aggregated substances which can’t distinguish the original form (Figure 3a,b) can be seen. The introduction of CNTs slightly weakened the degree of morphological damage, but it was hardly helpful to improve stability. However, the specific capacitance of NST/CNTs@C was only reduced from 620 F·g^−1^ to 512.5 F·g^−1^ in the first 20 cycles. That the morphology of NST/CNTs@C maintained almost unchanged after 20 cycles (Figure 6c) suggests that the carbon-coated layer had successfully restrained the volume expansion and contraction in the reaction process. The specific capacitance of all three electrode materials decreased at a relatively slow rate from the 20th to the 100th cycles and then tended to be stable after 100th cycles. The morphological changes of all three kinds of electrode materials were not obvious from the 20th cycle to the 1000th cycle (Figure 6d–f), which is why the specific capacitance decay rate slowed down. The retention rates of NiS_2_, NiS_2_/CNTs and NST/CNTs@C were 1.6%, 0.56% and 49.19% after 1000 cycles, respectively. The cycle stability of NST/CNTs@C had been tremendously improved.

**Figure 7:**
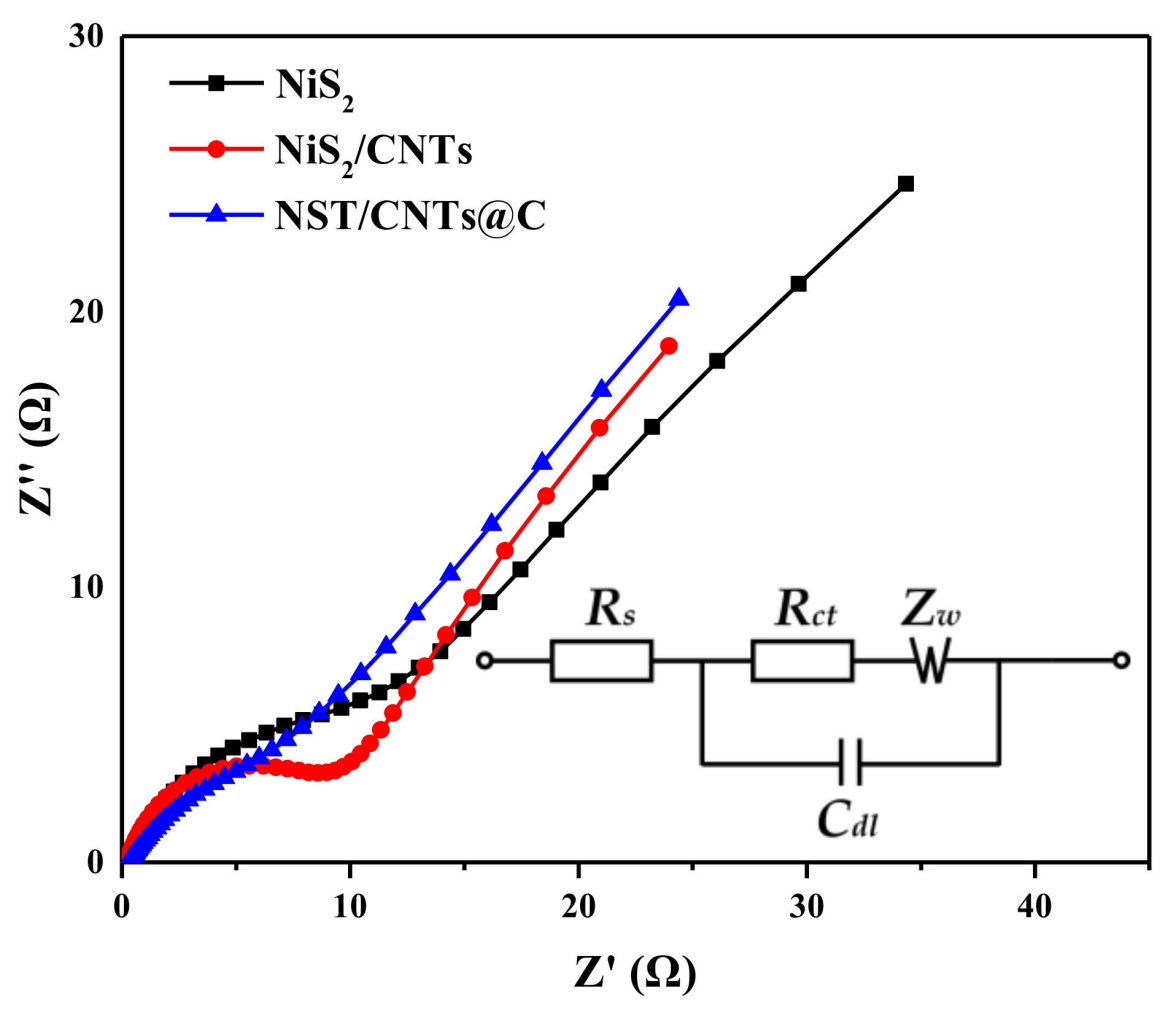
Nyquist plots of NiS_2_, NiS_2_/CNTs and NST/CNTs@C electrodes, the inset is equivalent circuit model.

The electrochemical performances were further explained by the electrochemical impedance spectroscopy (EIS) text. Figure 7 showed the Nyquist plots which obtained in the frequency range from 100 kHz to 0.01 Hz and the quivalent circuit model of three electrode materials. In general, the Nyquist plot of electrode materials for redox supercapacitors should include a semicircle related to Faradaic reactions in high-frequency region and a straight line related to Warburg impedance in low frequency region [58]. It can be seen from the equivalent circuit model, the electrode systems contained electrolyte solution resistance (*R_s_*), charge transfer resistance (*R_ct_*), Warburg impedance resistance in ions diffusion process (*Z_w_*) and the double layer capacitance at the electrode/electrolyte interface (*C_dl_*). The *R_ct_* of NiS_2_, NiS_2_/CNTs and NST/CNTs@C can be calculated to be 8.26 Ω, 7.37 Ω and 4.25 Ω, respectively. It clearly revealed that the electronic conductivity had been improved by the introduction of carbon materials. Furthermore, the electronic conductivity may be an influence factor of rate capability. The higher slope of the straight line indicated lower ions diffusion resistance and more outstanding capacitance performance [21]. The sequence of the slopes from low to high: NiS_2_, NST/CNTs@C and NiS_2_/CNTs. This result was consistent with the CV curves and the GCD curves.

## 4. Conclusions

In summary, we successfully synthesized a new carbon-coated nickel sulfide electrode material (NST/CNTs@C) by introducing two different forms of carbon materials, in which the specific capacitance was increased with the addition of CNTs and the rate performance, and cycle stability were improved by the carbon-coated layer which was formed with BTC as carbon source. The carbon-coated layer effectively enhanced the electrical conductivity and restricted the deformation of nickel sulfides in the process of GCD. Compared with pure NiS_2_, the specific capacitance of NiS_2_/CNTs raised from 430 F·g^−1^ to 885 F·g^−1^ at a current density of 1 A·g^−1^. And NST/CNTs@C also showed good cycling stablility with 49.19% capacitance retention at 1 A·g^−1^ after 1000 cycles (the capacitance retention of NiS_2_ and NiS_2_/CNTs were only 1.6% and 0.56%). Comprehensively, the introduction of CNTs and carbon-coated layer was an effective method to improve the overall electrochemical properties of nickel sulfides.

## Figures and Tables

**Table 1:** Comparison of specific capacitance, rate capability and stability of samples with different carbon sources.

Carbon Source	*C_m_* (F·g^−1^) (Current Density: 1 A·g^−1^)	*C_m_* (F·g^−1^) (After 1000 Cycles)	*C_m_* (F·g^−1^) (Current Density: 10 A·g^−1^)
BTC	620	305	275
PANI	535	12.5	25
Glucose	505	7.5	25
